# Educators’ perceptions of e-cigarettes in Australian secondary schools

**DOI:** 10.18332/tid/161025

**Published:** 2023-03-16

**Authors:** Michelle I. Jongenelis, Abby Robinson

**Affiliations:** 1 Melbourne Centre for Behaviour Change, Melbourne School of Psychological Sciences, The University of Melbourne, Melbourne, Australia

**Keywords:** e-cigarettes, secondary schools, educators, students, youth

## Abstract

**INTRODUCTION:**

Secondary schools are a setting in which e-cigarette use among students has increased significantly, resulting in an urgent need for educators to develop and implement strategies to curb youth vaping. Research assessing school-based vaping prevention efforts is limited and largely confined to the US. This study assessed Australian secondary school staff members’ experiences with e-cigarettes and explored (i) the presence of e-cigarette policies and educational programs, (ii) barriers to policy development and implementation, and (iii) desired support.

**METHODS:**

Public, Catholic, and Independent secondary schools across Australia were sent an invitation to participate in this study, which involved completion of an online survey. A total of 218 school staff members (55% women) participated. Respondents included school principals, teachers, and other staff members. Data collection occurred May to September 2022. Both quantitative and qualitative data were collected.

**RESULTS:**

Nearly half (46%) of all school staff members surveyed reported finding a student with an e-cigarette on campus at least monthly, and one-third (36%) of principals reported suspending or expelling students at least monthly for e-cigarette possession or use. The vast majority of those surveyed agreed that e-cigarette use is increasingly becoming a problem in secondary schools (93%) and reported being concerned about e-cigarette use by students (94%). Only half (51%) reported that their school had an e-cigarette policy in place. The discreet appearance of e-cigarettes (83%) and difficulties pinpointing from where the vapor/scent is coming (73%) were the most frequently reported barriers to policy enforcement.

**CONCLUSIONS:**

The results of this study suggest that e-cigarettes present a threat to secondary school environments. There is an urgent need to develop, implement, and enforce both school- and government-level e-cigarette policies to prevent and reduce youth vaping in Australian secondary schools.

## INTRODUCTION

Use of e-cigarettes (known colloquially as vaping) has increased significantly in Australia, especially among young people^1^. The rapid rise in use among youth is of concern given the adverse health effects associated with exposure to the toxicants in the aerosol generated by e-cigarettes^2-4^ and the link between e-cigarette use and subsequent initiation of tobacco smoking^4^. Efforts are thus urgently needed to halt the rising prevalence of vaping among youth.

Secondary schools are a setting in which e-cigarette use among students has increased significantly in the last decade, especially in countries such as the US and the UK^5-7^. Results from studies conducted in the US suggest on-campus use of e-cigarettes is common^8-10^, with most educators considering use on school grounds to be a problem and expressing concerns^11,12^. Research assessing Australian educators’ perceptions of e-cigarette use in secondary schools is limited, with just one study published to date^13^. Results from this study indicate e-cigarettes are a significant challenge in school settings, with the majority of school staff surveyed expressing concerns about the prevalence of use and its consequences among students.

The school environment plays an important role in the development of adolescent health behaviors^14-16^, including vaping^17^. Research from the US indicates that: 1) observing peer e-cigarette use on school campus doubles the odds of ever e-cigarette use and susceptibility to use^10^; and 2) students attending schools with medium and high prevalence of e-cigarette use are more likely than their counterparts at low-prevalence schools to use e-cigarettes more frequently and be willing to try e-cigarettes^17^. This suggests that use of e-cigarettes in secondary schools may contribute to the normalization of vaping among students and the development of a school environment that fosters engagement in harmful health behaviours^18^.

In recognition of the importance of the school environment and school staff in shaping students’ perceptions, schools are considered critical to the implementation of multilevel interventions that promote healthy behaviors and dissuade engagement in unhealthy behaviors^19,20^. Tobacco smoking has been the target of school-based interventions for decades^21^. The emergence of e-cigarettes has resulted in the need for schools to develop programs addressing the growing threat of vaping, with teachers identified as important stakeholders in efforts to curb youth e-cigarette use^22^. However, research assessing school-based vaping prevention efforts is limited and has largely been conducted in the US. This research found that school-level policies play an important role in preventing and restricting e-cigarette use^12,23^. For example, use is significantly lower at schools that have a policy prohibiting vaping on school grounds compared to schools without such a policy^23^. The presence of an e-cigarette policy has also been found to be associated with greater awareness of e-cigarettes among school staff and greater odds of school personnel communicating with students about avoiding e-cigarette use^12^. These findings underscore the importance of implementing school-based e-cigarette policies to reduce vaping among youth and support staff to communicate about e-cigarettes, both of which have the potential to change school-based norms^12^. Yet, most Australian schools do not have e-cigarette policies in place^13^.

The increasing use of e-cigarettes among Australian secondary school students warrants immediate action. As a group that has regular contact with a large number of children^13^, secondary school staff members are an important source of information on e-cigarette use among students. However, most research on student vaping has been conducted in the US, a country with a more lenient regulatory environment for e-cigarettes than Australia (which has prohibited the sale of nicotine-containing e-liquid outside the pharmaceutical model, but allows the sale of non-nicotine e-liquid). Data assessing the concerns of Australian educators in relation to e-cigarettes, and the extent to which the devices are perceived to be posing a problem in secondary schools, are limited. Crucially, research assessing barriers to policy development, implementation, and enforcement is lacking, making it difficult for principals, teachers, and policymakers to make informed decisions about how best to deal with e-cigarettes in secondary school settings. The present study aimed to address these substantial gaps in knowledge by assessing Australian secondary school staff members’ experiences with student e-cigarette use and exploring: 1) the presence of e-cigarette policies and educational programs; 2) barriers to policy development and implementation; and 3) desired support.

## METHODS

### Design

A cross-sectional online survey was administered May to September 2022. The survey collected both qualitative and quantitative information. This research was approved by the Human Research Ethics Committee of The University of Melbourne, and approval to approach secondary schools (which comprise some or all grades from 7 to 12), was sought from relevant authorities. Some authorities did not grant permission, citing the ongoing burden of COVID-19 on school personnel at the time of data collection. Accordingly, additional schools from the jurisdictions where approval was granted were sampled. With the exception of the Northern Territory, schools in all Australian States and Territories were represented.

### Recruitment

The Australian Curriculum, Assessment and Reporting Authority’s database was used to identify secondary schools in Australia. A total of 863 Catholic, Independent and Public schools from the jurisdictions where approval to conduct the study was granted were randomly selected to participate. Once selected, the principals of each of the schools were sent an email inviting them to complete an online survey. This email included a detailed information sheet describing the present study and encouraged principals to share the survey link with other staff members within their school. All respondents provided informed consent.

### Measures

Respondents were asked to provide basic demographic (e.g. gender, age) and school-related information (e.g. school type, school size, student profile). They were also asked to indicate their role within the school (principal, deputy principal, teacher, other; dichotomized into ‘Principals’ and ‘Other’ for analysis purposes). Prior to answering questions relating to e-cigarettes, respondents were provided with a description of e-cigarettes and informed of some names by which these devices are also known (e.g. vapes, Puff Bars).

*E-cigarette risk perceptions*

Questions were posed to assess the extent to which respondents agreed that: 1) nicotine and 2) non-nicotine e-cigarettes are harmful to health (1=strongly disagree to 5=strongly agree). Respondents’ overall opinion of e-cigarettes was also assessed (1=very negative to 5=very positive), as was their knowledge of whether it was legal for those aged <18 years to possess an e-cigarette (yes/no/don’t know).

*Possession and use of e-cigarettes and tobacco cigarettes in secondary schools*

Respondents were asked to report on the frequency (daily, 2–3 times per week, weekly, fortnightly, monthly, less often than monthly, never) with which they found students in possession of or using: 1) e-cigarettes and 2) tobacco cigarettes on school premises over the previous 12 months. School principals were additionally asked to report on how often (daily, 2–3 times per week, weekly, fortnightly, monthly, less often than monthly, never) they suspended or expelled students for: 1) using e-cigarettes and 2) smoking tobacco cigarettes on school premises over the previous 12 months. Principals were also asked to provide an approximate number of e-cigarette devices confiscated over the same time period (open response option).

*Perceptions of e-cigarette use in secondary schools*

Respondents were asked two questions assessing the extent to which they agreed that e-cigarette use is becoming a problem in secondary schools generally, and in their school specifically (1=strongly disagree to 5=strongly agree). They were also asked to report on the extent to which e-cigarette use by students on school grounds had been a problem in their school over the previous 12 months (1=not a problem to 4=very serious problem) and how concerned they were about e-cigarette use by students at their school (1=not at all concerned to 4=very concerned). Those who reported being at least somewhat concerned were subsequently asked to describe their concerns (open response). This section of the survey ended with questions asking school staff members how much of a priority is addressing e-cigarette use at their school (1=not at all a priority to 6=very high priority) and how confident they are in their ability to: 1) detect and 2) address e-cigarette use among students (1=not very confident to 4=very confident).

*School policies and education*

Questions were posed that assessed: 1) school policies regarding e-cigarette and tobacco use (i.e. presence of such policies; barriers to implementation and enforcement, as per Schillo et al.^11^); 2) measures adopted to manage e-cigarette use (e.g. installation of vaping detectors); and 3) the presence of education about e-cigarette and tobacco use. If policies existed in their school, principals were asked to describe these briefly. If there were no school policies, principals were asked to provide an explanation for their absence. Finally, school staff members were asked to indicate any desired support, information, and resources they believed would assist them to manage e-cigarette use (open response).

### Statistical analysis

Descriptive statistics were calculated in SPSS for the various measures collected (e.g. risk perceptions, extent of e-cigarette use among students, concerns about use). Means with standard deviations and proportions are presented. Responses to open-ended questions were subject to reflexive thematic analysis in NVivo. As is customary with this form of analysis, a single researcher coded all responses^24^. Example quotes are provided to illustrate key points.

## RESULTS

### Sample

A total of 218 school staff members completed the survey. Characteristics of the respondents and their schools are presented in Table 1. As the survey was anonymous, it is unknown how many individual schools participated. However, exploration of postal code data suggests at least 101 unique schools were represented.

**Table 1:** Participant and school characteristics, Australia, 2022 (N=218)

*Characteristics*	*n*	*%*
**Gender**		
Female/woman	121	55
Male/man	94	43
Non-binary	2	1
Other	1	1
**Age** (years), Mean (SD)	44.99 (10.64)	
**Role**		
Principal	42	19
Other (e.g. teacher, administrator, counsellor)	176	81
**Education level**		
Secondary only	165	76
Combined Primary/Secondary	53	24
**School type**		
Catholic	93	43
Independent	67	31
Public	58	27
**Student demographic**		
Co-educational	177	81
All boys	34	16
All girls	7	3
**Approximate number of students in grades** ≥**7** Mean (SD)	808 (616)	
**Location**		
Metropolitan	126	58
Regional	84	39
Rural/remote area	6	3
Missing	2	<1

### E-cigarette risk perceptions

The vast majority of secondary school staff members surveyed agreed that e-cigarettes with (98%; Mean score=4.86, SD=0.59) and without (93%; Mean score=4.50, SD=0.83) nicotine are harmful to health. The vast majority also reported having a negative opinion of the devices (95%; Mean score=1.37, SD=0.63). Three-quarters (75%) accurately reported that it was illegal for those <18 years of age to possess an e-cigarette, 9% incorrectly reported that it was legal, and 16% did not know.

### Possession and use of e-cigarettes and tobacco cigarettes in secondary schools

Nearly half (47%) of all school staff members surveyed reported finding a student with an e-cigarette at least monthly, and nearly one-quarter (24%) reported finding a student with an e-cigarette at least weekly (Table 2). Fewer reported finding a student with a tobacco cigarette (16% at least monthly, 9% at least weekly). Reports from the school principals surveyed indicate suspensions or expulsions for tobacco cigarette smoking were infrequent (7% at least monthly, 2% at least weekly). By contrast, approximately one-third (36%) of principals reported suspending or expelling students at least monthly for e-cigarette possession or use, and 12% at least weekly.

**Table 2:** Possession or use of e-cigarettes and tobacco cigarettes, Australia, 2022

	*n*	*%*
**Finding students in possession of or using e-cigarettes**		
Daily	8	4
2–3 times per week	23	11
Weekly	20	9
Fortnightly	23	11
Monthly	27	12
Less often than monthly	58	27
Never	59	27
**Finding students in possession of or using tobacco cigarettes**		
Daily	5	2
2–3 times per week	9	4
Weekly	7	3
Fortnightly	3	1
Monthly	14	6
Less often than monthly	49	23
Never	131	60
**Suspension/expulsion for possession or use of e-cigarettes^a^**		
Daily	0	0
2–3 times per week	2	5
Weekly	3	7
Fortnightly	3	7
Monthly	7	17
Less often than monthly	17	40
Never	10	24
**Suspension/expulsion for possession or use of tobacco cigarettes^a^**		
Daily	0	0
2–3 times per week	0	0
Weekly	1	2
Fortnightly	0	0
Monthly	2	5
Less often than monthly	10	24
Never	29	69
**Number of e-cigarettes confiscated^a^**		
Mean (SD)	18.62 (25.24)	
Range	0–100	

Due to rounding, figures may not add up to 100%.

a Only principals were asked to report on suspensions/expulsions and confiscated devices.

### Perceptions of e-cigarette use in secondary schools

Results relating to school staff members’ perceptions of e-cigarette use in secondary schools are presented in Table 3. The vast majority of staff members surveyed agreed that e-cigarette use is increasingly becoming a problem in secondary schools generally (‘strongly agree’=67%, ‘somewhat agree’=26%) and in their school specifically (‘strongly agree’=44%, ‘somewhat agree’=39%). The vast majority also reported that e-cigarette use on school grounds is becoming at least ‘somewhat’ of a problem (‘very serious problem’=22%, ‘moderately serious problem’=38%, ‘somewhat serious problem’=31%) and were at least ‘somewhat’ concerned about e-cigarette use by students at their school (‘very concerned’= 43%, ‘moderately concerned’=34%, ‘somewhat concerned’=17%). Most reported that addressing e-cigarette use was a priority and had the confidence to do so; however, fewer reported having the confidence to detect e-cigarette use among students.

**Table 3:** Perceptions of e-cigarette use in secondary schools, Australia, 2022 (N=218)

*Perceptions*	*Mean score (SD)*	*%f*
Use is increasingly becoming a problem in secondary schools generally^a^	4.57 (0.74)	93
Use is increasingly becoming a problem in their school specifically^a^	4.10 (1.09)	83
Extent to which use on school property has been a problem^b^	2.71 (0.92)	90
Concern about e-cigarette use by students at their school^c^	3.15 (0.90)	94
Priority to address e-cigarette use^d^	4.42 (1.26)	80
Confidence to address use among students^e^	2.38 (0.94)	82
Confidence to detect use among students^e^	1.95 (0.86)	66

a Responses made on a scale of 1 (strongly disagree) to 5 (strongly agree).

b Responses made on a scale of 1 (not a problem) to 4 (very serious problem).

c Responses made on a scale of 1 (not at all concerned) to 4 (very concerned).

d Responses made on a scale of 1 (not at all a priority) to 6 (very high priority).

e Responses made on a scale of 1 (not very confident) to 4 (very confident).

f Proportion who responded somewhat agree/strongly agree; somewhat serious problem/moderately serious problem/very serious problem; somewhat concerned/moderately concerned/very concerned; medium priority/somewhat high priority/very high priority; or somewhat confident/moderately confident/very confident.

Reflexive thematic analysis was used to explore staff concerns around student e-cigarette use. The most common concerns raised by respondents related to: 1) the health risks associated with use and the potential for addiction in students (43%); 2) the ease with which use can be hidden, making it difficult to detect (18%); 3) students’ lack of awareness of the harms associated with use (15%); 4) use in toilets, creating an unsafe environment (14%); and 5) the increasing number of younger students using e-cigarettes (12%). Other concerns raised, albeit less frequently, included the ease with which e-cigarettes can be accessed by students (8%), students selling e-cigarettes to each other (8%), e-cigarette use as a gateway to smoking and other drug use (8%), and students missing classes to engage in e-cigarette use (5%):

*‘I’m concerned regarding the addictive nature of e-cigarettes, the link to future tobacco smoking, and the health impacts of both actions.’* (Female, teacher, Independent school)

*‘Students using e-cigarettes at school, not sure where they are sourcing these from so their contents is not controlled/safe, their use in toilets/bathrooms at school make other students feel unsafe about using these spaces.’* (Female, senior leader, Public school)

*‘I have spoken with many 14-year-olds who claim to be addicted/unable to control their desire to use them. They are having a significant impact on the health of students.’* (Male, deputy principal, Public school)

*‘… that students believe they are harmless. They are marketed and sold as if they are confectionery. Students believe that vaping and eating sweets equates to the same thing.’* (Male, deputy principal, Catholic school)

### School policies and education

Just over three-quarters of respondents (78%) reported that their school had a tobacco smoking policy, whereas only half (51%) reported that their school had an e-cigarette policy. Among principals who reported that their school did not have an e-cigarette policy (n=25), the most common reason given was that vaping was captured under other school policies; for example, drug or suspension policies (n=14; 56%). Other reasons for the absence of an e-cigarette policy included: 1) use not yet being an issue (32%) and 2) lack of time (16%). Among principals who reported that their school had an e-cigarette policy (n=17), e-cigarettes were considered a prohibited product by all. Just over half (53%) reported that the policy involved disciplinary action (e.g. suspension). Almost half (47%) reported that the policy involved educating students and/or required that students caught with e-cigarettes meet with the school nurse.

Among staff members who reported that their school had an e-cigarette policy (n=111), the most frequently nominated barrier to enforcement was that e-cigarette products are discreet in appearance (83%), followed by difficulties pinpointing from where the vapor/scent is coming (73%). Some school staff members reported that parents do not support the policy (11%). Few reported a lack of clarity about the policy (5%) and how it should be enforced (3%).

In terms of education, most respondents (88%) reported that their school educated students on tobacco use. Slightly fewer (77%) reported that their school educated students on vaping. When asked about other approaches to the management of e-cigarette use among students, 19% of respondents reported that their school had installed vaping detectors (49% had considered installing these), 18% reported that their school educated parents about e-cigarettes, 10% reported monitoring students for use, and 6% reported educating teachers. In terms of desired support, 50% of school staff members reported desiring education programs for students and staff members and 22% desired the installation of vaping detectors. Some desired: 1) information on how to detect use (7%) and 2) greater parental involvement (6%).

## DISCUSSION

As a setting in which adolescents spend a substantial proportion of their time, the school environment has the potential to play an important role in combating the rise in the use of e-cigarettes among youth. The present study sought to assess Australian secondary school staff members’ experiences with e-cigarette use among students and explore school-based e-cigarette policies and educational programs. The results provide information about e-cigarette use in secondary schools and identify means by which schools can be supported; information that can be used to develop relevant and evidence-based resources, programs, and policies that assist those who are well placed to protect young Australians from establishing harmful habits in their formative years.

Consistent with previous research^13^, the results of the present study suggest that e-cigarettes are a threat to Australian secondary school environments. Nearly half of the staff members surveyed reported finding students with an e-cigarette at least monthly and around one-third of principals reported that they had suspended or expelled students at least monthly for e-cigarette possession or use. While fewer respondents reported observing possession or use of tobacco cigarettes, it is still concerning that nearly one-fifth reported finding students in possession of or smoking tobacco cigarettes at least monthly. These findings suggest that Australia’s tobacco control efforts must be accelerated and that renewed attention to reduce smoking rates is urgently needed. Little has been done in relation to tobacco cigarette smoking since the introduction of plain packaging a decade ago, with experts calling for increased investment in measures such as national mass media campaigns, updated warnings on tobacco packaging, and the expansion of smoke-free areas^25^. These measures serve to denormalize smoking and have been found to be effective at reducing smoking initiation among adolescents^26,27^.

Given the high frequency with which school staff members reported observing e-cigarette use among students, it is not surprising that the vast majority of those surveyed believed use was increasingly becoming a problem in secondary schools and expressed concerns about vaping among students. Despite this, only half reported that their school had an e-cigarette policy in place. Evidence indicates that school-level policies play an important role in preventing and restricting e-cigarette use^23^ and have the potential to change school-based norms^12^. The results of the present study suggest that there is an urgent need for schools and their associated education authorities to develop and implement targeted e-cigarette policies or amend existing tobacco control policies to include e-cigarettes.

Most respondents reported that addressing e-cigarette use was a priority at their school, suggesting that schools are motivated to implement vaping control policies, but may require assistance with policy development. For example, one-third of principals reported suspending or expelling students at least monthly for e-cigarette use, yet previous research in the context of tobacco smoking has found that the presence of sanctions is either not associated with student smoking or may contribute to increased smoking risk^28^. It is thus important that schools are encouraged to adopt approaches that are not punitive in nature. Rather, communicating to schools that policies are most effective when they feature prevention education and are comprehensive and clear^28^ is critical to ensuring any developed policies have the potential to impact vaping rates.

School policies are more likely to be effective when they are consistently enforced^28^. Results of the present study suggest that schools may require support with this aspect of policy implementation, with several barriers to enforcement cited by those staff members who reported that their school had an e-cigarette policy in place. The most frequently reported barriers were: 1) the discrete appearance of e-cigarette products and 2) difficulties pinpointing from where the e-cigarette vapor is coming. Such barriers have also been identified in research conducted in the US^11^ and likely explain why the proportion of staff members in the present study who expressed confidence in their ability to detect e-cigarette use among students was substantially lower than the proportion who expressed confidence in their ability to address use. With the proliferation of product innovations that make it easier for users to engage in ‘stealth vaping’^29^, stronger regulations of e-cigarettes are needed to minimize the extent to which users are able to avoid detection and flout smoke-free policies.

Although the majority of respondents noted that their school provided education on e-cigarette use, half reported desiring education programs for students and staff, and some reported desiring programs for parents. Several health organizations (e.g. Lung Foundation Australia, Quit, NSW Health) have developed evidence-based toolkits for educators that may assist them to manage e-cigarette use among youth. It is critical that members of the school community, including parents, be made aware of these resources to reduce any burden associated with schools developing their own interventions. Ensuring staff members are adequately trained to deliver any interventions is also essential to implementation. Past research in the context of tobacco smoking suggests training that: 1) targets teachers’ motivation and self-efficacy to deliver an intervention and 2) provides teachers with knowledge of their responsibilities and the information and skills needed to successfully implement the intervention may enhance implementation effectiveness^30^.

Finally, it must be noted that comprehensive, multilevel efforts are needed to address e-cigarette use among secondary school students^11^. While schoolbased approaches are important, the Australian Government must also act to reduce the ease with which e-cigarettes can be accessed by school children, and government-led policies need to be introduced that reduce the accessibility and availability of e-cigarettes to minimize the burden being placed on schools to manage vaping among students. Recent research examining access to vaping products among adolescents aged 14–17 years in New South Wales, Australia, found that the majority reported it was easy to access the products, with nearly one-third of those who had purchased their own e-cigarette doing so from a retailer^31^. Prohibiting the sale and importation of all e-cigarettes and related components outside the Therapeutic Goods Administration's pharmaceutical scheme is recommended to reduce the availability and accessibility of these products. This includes non-nicotine products, which are harmful to health^32-35^ and can act as a Trojan Horse for the importation and sale of nicotine products. Greater enforcement of existing laws that prohibit the sale of nicotine e-cigarettes is also urgently needed.

### Limitations

This study had several limitations. First, staff members from Catholic and Independent schools were over-represented in the sample. This is likely to be at least partially due to the authorities of some public school jurisdictions declining to participate, citing the burden of COVID-19 on school personnel. Second, as multiple personnel from within schools were invited to complete the anonymous survey, it is unknown how many unique schools were represented. Third, although schools were randomly selected to participate, completion of the survey was voluntary and respondents thus self-selected into the study. In addition, as principals were asked to forward the link to the study survey to staff members, a convenience sample of teachers and other staff members was surveyed. Given the potential for selection bias, results may not be generalizable to all Australian secondary school educators. Fourth, comparisons between school staff members’ reports of e-cigarette use and students’ reports of e-cigarette use could not be made as data relating to the latter were unable to be collected due to COVID-19 restrictions. Finally, the data on school expulsions is limited, as some Australian jurisdictions collect this information at the authority-level and not at the school-level. The results presented here may therefore underestimate the extent to which students are suspended or expelled for vaping and smoking.

## CONCLUSIONS

The high frequency with which school staff members surveyed in the present study reported observing e-cigarette use among students suggests efforts are urgently needed to address the growing use of e-cigarettes among school children. The development, implementation, and enforcement of both school- and government-level e-cigarette policies to prevent and reduce youth vaping in Australian secondary schools is warranted.

## Data Availability

The data supporting this research are available from the authors on reasonable request.
